# Engaging the next generation: authoritarian regimes and their young diaspora

**DOI:** 10.1057/s41304-022-00409-2

**Published:** 2023-01-25

**Authors:** Arne F. Wackenhut, Camilla Orjuela

**Affiliations:** School of Global Studies, University of Gothenburg, Box 700, SE 405 30 Göteborg, Sweden

**Keywords:** Second-generation diaspora, Migrant descendants, Transnational repression, Diaspora engagement, Diaspora activism

## Abstract

Recent scholarship on diaspora engagement and transnational repression has investigated how authoritarian regimes seek to engage, govern and control their diasporas. Recognizing that diasporas are diverse and that homeland states thus devise different strategies in relation to different groups, this research has—to a large extent—focused on the varied positions held by regime supporters and dissidents. Inter-generational differences, however, have not been studied in this context. Drawing on established frameworks theorizing extraterritorial authoritarian practices, this article explores the ways in which second-generation diaspora—or diaspora youth—is either included as subjects, patriots and clients, or excluded as outlaws and traitors by authoritarian regimes. Drawing on the literature on transnationalism and second-generation migrants, and using examples from empirical cases, we argue that the skills, resources and multi-sited embeddedness of the second-generation diaspora can make them particularly interesting targets for transnational engagement—or repression. We draw attention to specific strategies for mobilizing the support of diaspora youth, but also note that some techniques to control or repress extraterritorial subjects are less efficacious in relation to this generation.

## Introduction

It is well known that authoritarian regimes engage in transnational repression—including surveillance, unlawful renditions, harassment, and even assassinations—to silence critics in the diaspora (Glasius 2017; Moss 2016; Schenkkan and Linzer 2021). However, the recent revelation concerning Carine Kanimba, daughter of exiled Rwandan dissident Paul Rusesabagina—globally known as the hotel manager who saved over a thousand lives during the 1994 genocide—surprised some observers. She was among the many human rights activists, journalists, and political opponents whose phones had been compromised by the Rwandan government. The fact that a US–Belgian citizen, who had not set foot in Rwanda since she left as a small child, would be targeted, illustrated with shocking clarity the long reach of authoritarian regimes (OCCRP 2021).

Scholars have started to analyse the ways in which authoritarian governments seek to repress, but also to collaborate with or co-opt their dispersed populations (see e.g. Moss et al. 2022; Tsourapas 2021). The targeting of young diasporans like Kanimba shows how this diaspora engagement not only transcends geographical borders but also generations. Authoritarian regimes, indeed, not only encourage their first-generation diaspora^1^ to contribute to development and image-building for the homeland, but also seek to engage young (and/or) second-generation^2^ diasporans through invitations to invest in, “return” to, or learn about their country of origin in various ways (c.f. Abramson 2017; Mahieu 2019). Simultaneously, second-generation diaspora activists criticize authoritarian regimes and mobilize for human rights and democratization, making use of their position as integrated and educated in democratic countries (Orjuela 2020).

Diasporas are vastly diverse and studies of regime–diaspora relations always run the risk of treating them as monolithic entities. While earlier research has highlighted the different positionalities of regime supporters and dissidents in the diaspora (c.f. Baeza and Pinto 2016; Turner 2013), potential differences between generations have so far been largely neglected. To begin filling this gap in the extant literature, we explore the extent to which authoritarian regimes engage with or seek to repress their second-generation diasporas, and how the special positionality of this generation shapes such state-diaspora relations.

First-generation migrants are likely to have close links to and family in the authoritarian countries they left, and they often struggle with issues of integration and their potentially precarious immigration status as non-citizens. Their children are more likely to live their lives further away from the tentacles of their parents’ authoritarian homeland states—but may nevertheless identify and seek to engage with it. To better understand the positionality and special characteristics of the second-generation diaspora in this context, we draw on the diverse literatures on transnationalism and second-generation migrants within an interdisciplinary framework. Relying on Glasius’ (2017) framework of extraterritorial authoritarian practices, we weave together the quite fragmented and largely single case study-based literature on authoritarian regimes’ engagement with their second-generation diasporas to shed light on the ways in which second-generation diasporans are either included as subjects, patriots, and clients, or excluded as outlaws and traitors by their ancestral homeland states. This means that both our ambition and contribution are chiefly conceptual rather than empirical: we draw attention to a population that—so far—has been largely overlooked in studies on authoritarian diaspora engagement.

We find, most importantly, that the specific positionality and activism of the second generation have crucial implications for the practices that authoritarian rulers employ to engage, govern, but also repress their non-resident populations. As the introduction of this special issue illustrates (Umpierrez de Reguero and Peltoniemi 2023), there are manifold ways to connect non-residents with specific territories. In this article, thus, we aim to elucidate how second generations and the origin countries of their ascendents may be intertwined within an authoritarian context.

For this purpose, we proceed as follows: after briefly reviewing earlier research on the ways in which authoritarian regimes seek to maintain stability and strive to either engage or control their diasporas, we pivot towards the second generation and discuss its specific positionality in relation to an ancestral homeland and its regime. Against this backdrop, we tease out the primary ways in which regimes seek to selectively include or exclude second-generation diasporans. In a concluding section, we summarize our key findings, address some of the broader implications and point towards avenues for further research.

### Authoritarians and their (transnational) subjects: an overview

Authoritarianism is surging, while democracy is in retreat. In 2020, “[f]or the first time since 2001, autocracies are in the majority: 92 countries—home to 54% of the global population” (Lührmann et al. 2020). Scholarly interest in understanding the inner workings of non-democratic regimes has paralleled this empirical trend. Traditionally, processes and dynamics *within* the territorial borders of authoritarian polities have been the main focus for inquiries in this space (see e.g. Frantz and Kendall-Taylor 2014; Sá and Rodrigues Sanches 2021; Wintrobe 2000). Gerschweski (2013) suggested, for instance, that the stability of authoritarian regimes essentially rests on three interdependent and mutually reinforcing pillars: varying configurations of *repression*, *co-optation*, and *legitimacy*. These pillars help to explain the (in-)stability of particular political orders and allow for a processual or interactionist account of the ways in which the three might (de-)stabilize a regime over time.

Recently, the transnational space has emerged as another important piece of the regime stability puzzle. Authoritarians are increasingly confronted with populations that are no longer confined to well-delineated national territories, but dispersed across space and borders (Glasius 2017; Tsourapas 2021). People emigrate to pursue economic opportunities, education, join family members, marry, or to seek safety and freedom of expression. Even while settling in their new countries, they often maintain transnational links and diasporic networks, and can therefore remain relevant actors in their countries of origin. Not least due to these transnational linkages, it has become a priority for migrant-sending states—both democratic and non-democratic—to harness the support of their diasporic populations. For authoritarian regimes, it is furthermore important to mitigate the threat that an unruly diaspora might pose.

States have increasingly devised diaspora engagement policies, which often include the establishment of dedicated government institutions, provisions to vote and hold dual citizenship, investment schemes, tourism initiatives or diaspora conferences (c.f. Pedroza and Palop-García, 2017; Ragazzi 2014). Authoritarian regimes have adapted their domestic tool kits to leverage diasporas as a resource and alleviate them as a threat. Here, the underlying logic resembles that of operating in the domestic setting. Even in the transnational space, the three pillars of legitimation, co-optation and repression can form the foundations of authoritarianism (c.f. Adamson 2020; Tsourapas 2021). Considering the diversity of non-resident populations in terms of their internal composition, their interests and attitudes, Glasius (2017) suggested that it might be fruitful to conceptualize the approach authoritarian regimes adopt vis-à-vis their extraterritorial populations in terms of selective inclusion and exclusion. She argued that:

the authoritarian state includes its citizens as subjects (to be controlled and repressed), as patriots (getting them to buy into legitimation strategies) or as clients (with potential for co-optation). When populations abroad resist being included in these ways, they may be excluded, and treated as outlaws (denied any trappings of legal personality) and/or as traitors (castigated and scapegoated as enemies of the state) (Glasius 2017).

Diasporic communities are important and therefore worthy of inclusion for at least five reasons. Firstly, through economic remittances, they are an important financial resource for the sending states and their populations. Secondly, direct investments from or the return of skilled emigrees to the sending states might stimulate economic development. Both temporary and permanent migration can, thirdly, act as a demographic safety valve that helps relieve pressures on the domestic labour market by exporting excess labour (Tsourapas 2015). Fourthly, harnessing the support of extraterritorial populations has become a priority for regimes for purely domestic political purposes (Brand 2010), as diaspora communities can shore up support for and legitimize the domestic political order by casting their votes and/or supporting election campaigns (cf. Umpierrez de Reguero and Jakobson 2023 in this special issue; Rashkova 2020; Wellman 2021). Lastly, regimes may also seek to utilize their extraterritorial populations as vehicles to advance their foreign policy interests (cf. Brinkerhoff 2019) through lobbying efforts, public protests, or media campaigns (cf. Mencutek and Baser 2018; Tsourapas 2016).

Still, under certain circumstances, diasporic populations might be seen as more of a liability if not a direct threat to authoritarian homeland governments. Migrant-receiving countries may provide space not only as a place of refuge, but also as platforms for mobilization and protests against repressive and violent homeland regimes (Betts and Jones 2016). A vast literature on “diaspora homeland politics” has analysed, for example, how migrant groups may attempt to influence political developments in what they still perceive to be their homelands (Lyons and Mandaville 2012; Orjuela 2008; Østergaard-Nielsen 2001).

Those who cannot or do not want to be *included* by their homeland regimes are therefore often subjected to a variety of different *exclusionary* practices (Glasius 2017). In some cases, this exclusion might be expressed through a hostile rhetoric. Discussing the case of Eritrea, Conrad (2006) noted, for instance, how the regime requires loyalty from its extraterritorial population and tends to brand open critique as mutiny or treason (see also Hirt and Saleh Mohammad 2018). Such hostile rhetoric is, however, just one end of a much broader spectrum of transnational repression. Authoritarian regimes have developed a sizable toolbox of strategies and practices to pursue their challengers and silence critique beyond their own borders, which includes assassinations, assaults, disappearances, renditions and unlawful deportations, abuse of Interpol’s system of “red notices”, digital threats, spyware, passport and document control, and coercion by proxy (Moss et al. 2022; Schenkkan and Linzer 2021; Tsourapas 2021).

So far, we have established that the stability of authoritarian regimes essentially rests on three interdependent and mutually reinforcing pillars—legitimation, co-optation, and repression. Furthermore, we have explored how regimes selectively include those parts of their diaspora that are deemed desirable or a resource. At the same time, those deemed undesirable, or a threat, are excluded, and the target of transnational repression. As we now turn to the question of how the second-generation diaspora fits into this equation, we rely on Glasius’s (2017) framework to explore how authoritarian regimes treat the second-generation diasporas as subjects over whom they can assert control, as patriots who can be mobilized to boost legitimacy, or as clients to be co-opted. Further, we also question whether this generation can be meaningfully excluded as outlaws or traitors, as authoritarian regimes strive to maintain stability and control. First, however, we survey the special positionality of the second-generation diaspora in relation to their ancestral homeland.

### The second-generation, or young, diaspora and the “homeland”

Who are the targets of transnational legitimation, co-optation, and repression? Non-resident citizens are often clear-cut cases, but what about their children or even grandchildren? Indeed, authoritarian regimes have different ideas of who is to be engaged, co-opted, controlled, or repressed. China, for instance, has “a broad and ever-expanding definition of who should be subject to extraterritorial control,” which includes allegedly corrupt former officials, entire ethnic and religious groups, as well as individuals of Chinese descent who are not citizens (Schenkkan and Linzer 2021). As of late, Turkey employs a similarly broad conceptualization of *its* diaspora, which covers émigrés, their descendants, and groups with historic ties to the Ottoman empire (Arkilic 2021). In the Rwandan case, all “Rwandans” abroad are at risk of transnational repression (Schenkkan and Linzer 2021)—potentially including both non-citizens and second-generation migrants. While few authoritarian regimes have a specific definition of and official policy directed towards migrant descendants, this group is often treated as co-nationals or transnational subjects that can be included or excluded.

Research on second-generation diasporans (including those whose ancestral homeland states are not necessarily autocratic) shows that this generation often maintains links to their relatives’ countries of origin while simultaneously being grounded in their country of residence. This means that they are raised in a transnational social field, which provides them with contacts and social skills that can be used in both settings. “They master several cultural repertoires that they can selectively deploy in response to the opportunities and challenges they face” (Levitt 2009: 1226). Analysing processes of identity construction and civic engagement among Somali diaspora youth, Horst (2018) noted how growing up in this transnational space often results in a multi-sited embeddedness—a sense of belonging to (and involvement in) multiple different locations in tandem. However, this multi-sited embeddedness entails its own challenges. Levitt (2009: 1230) remarked that second-generation migrants “have to make sense of at least two, often conflicting, socio-economic and racial stratification systems.” Discussing the experiences of Indian-American youth, she (2009: 1234) noted how parents “want their kids to fit in but not too much. The line between being ‘too American’ and ‘too Indian’ is never clear.” The notion of *in-betweenness* is often used to describe how second-generation migrants straddle between two worlds without necessarily feeling completely at home or welcome in either of them—a dynamic shown among, for instance, American-Egyptians. Saey and Skey (2016) found that, while this ambiguous identity can be troubling, second-generation migrants “returning” to Egypt selectively emphasized either their Egyptian or “foreign” identities depending on what helped them most in a particular situation.

While first-generation migrants may share the sense of not *fitting in* in either place, second-generation migrants tend to be more firmly embedded in their country of residence. A better education, language skills, and potentially improved economic prospects compared to first-generation migrants (see e.g. Zhou 1997), are likely to provide them with political and economic opportunities that can be harnessed for homeland engagements. Simultaneously, second-generation diasporans tend to have weaker transnational ties than the first generation (see e.g. Bloch and Hirsch 2018; Huang 2021; Soehl and Waldinger 2012). Tongan second-generation migrants have, for instance, fewer and less intense transnational links than their parents, which expresses itself in, for example, a notable drop in remittances being sent to Tonga from generation to generation (Lee 2011).

Whether second-generation diasporans develop an interest in their ancestral homeland and either mobilize support for or resistance against its regime depends on a range of factors. These might include special events in the parents’ country of origin, the situation in the country of residence, and/or relations to others in the diaspora. A crisis, such as a natural disaster or an armed conflict, in the ancestral homeland can mobilize the second-generation diaspora (see e.g. Orjuela 2020). The Egyptian uprising of 2011, for instance, triggered a “process that led to renewed interest in their parents’ country of origin” among numerous Austrian-born younger Egyptians (Lamblin 2018; Müller-Funk 2016: 363–364).

Inter-generational relations in the family also play a key role in engaging—or distancing—second-generation individuals from their parents’ country of origin. Research has shown how parents’ traumatic experiences might affect the life of younger generations, and how long-distance nationalism can provide meaning to both parents and children (see e.g. Bloch 2018; Bruland 2012; Fouron and Glick- Schiller 2002). In this context, Hirsch (2008: 106) discusses “post-memory” as “a structure of inter- and trans-generational transmission of traumatic knowledge and experience”. Although individuals of the next generation lack their own recollections of terrible events, the stories, and silences they have grown up with may have strong affective effects on them and serve as a motivation to engage in memorialization and activism. The descendants of victims might be driven by anger at the historical injustices and seek redress for what happened to their parents—or they may avoid engagement with their troublesome past altogether. Discussing the case of Turkish Kurds, Bloch and Hirsch (2018:15) noted how “for some, politics was simply inherited, passed on from parents to their children where there was an unquestioned expectation that they would be involved.” In other cases, parents dissuade their children from engaging politically with their former homeland for fear of repercussions.

Additionally, the host countries’ political systems and immigration and integration policies may also play a role in determining the degrees to which second-generation diasporans direct their attention and political engagement towards their parents’ countries of origin (Østergaard-Nielsen 2001). Migrant descendants are often less affected by immigration policies but may experience similar economic, social and political marginalization and racialization as their parents or grandparents (c.f. Barwick and Beaman 2019).

The simultaneous sense of (non-)belonging to several sites and the capacity to navigate different social and political contexts characterizes the migrant experience. Still, it can be especially pronounced for those of the second generation. This generation’s embeddedness in the countries they live in, their language skills, citizenship and other capacities and capabilities make them an interesting target for states seeking to engage their diaspora. Compared to first-generation migrants, they need to be more actively socialized into supporting and identifying with their ancestral homeland. Meanwhile, their positionality and resources may render them even more threatening if they turn against the non-democratic “homeland” regime. Thus, authoritarian regimes have strong incentives to include this group in their attempts to engage and/or control “their” diaspora—but they may meet particular challenges in the process.

### How do homeland regimes include the second-generation diaspora?

Some diaspora engagement strategies are particularly relevant for or explicitly geared towards second-generation migrants. Key strategies for engaging this target group include government-linked diaspora organizations that often have their own youth chapters and youth activities, as well as special conferences for young people organized in the diaspora. Homeland visits, organized by the state, diaspora organizations or through international collaborations, are yet another common activity designed to socialize this group into identifying with and supporting their ancestral homeland.

Rwanda, for instance, actively targets the second-generation diaspora and diaspora youth across Europe. The government arranges regular diaspora meetings, both in Rwanda and European countries, providing opportunities for diasporans to meet high-level representatives from Rwanda. Since 2008, the Rwandan government has a yearly five-week civic education programme, *Indangamirwa* (‘role model’) for youths between the ages of 18 to 35 who live or plan to study abroad. Participants learn about Rwandan history and get a chance to reconnect with their culture and language. Through other programmes, such as the *Rwanda Youth Tour*, diaspora youth have been invited to visit the country. Considering such activities, the Rwandan government clearly sees diaspora youth as a resource to be harnessed. The programmes encourage the participants to get involved in business and to use their skills in support of Rwanda’s development. As “ambassadors”, it is their task to help transform Rwanda’s image from a country primarily associated with the 1994 genocide to an attractive tourist destination and a self-sustaining and proud African state with a successful track record of post-genocide reconciliation and economic development. In July 2020, the Rwandan embassy in Sweden organized an online Liberation Day celebration and invited diaspora youth from all over the world to listen to and interact with, among others, Rwanda’s Ministers of Youth and Foreign Affairs. A young entrepreneur who had grown up in the diaspora but moved “back” to Rwanda was also among the invited speakers: embodying an exemplary patriotic and skilful young diasporan who supports his country. The many initiatives of this sort, and the active involvement of dignitaries such as ministers and the president himself, suggest that the Rwandan government prioritizes inclusion of its young/second-generation diaspora—involving them as patriots who can convey a “correct” image of Rwanda globally, and as clients who can support economic development.

Turkey is another excellent example in this space. Especially since the AKP rose to power in 2002, the Turkish government has prioritized an active engagement with its sizable diaspora, which encompasses more than 6 million Turkish citizens abroad—many of whom reside in European countries like Germany or the Netherlands. While the relative importance of remittances for the Turkish economy has shrunk markedly over the years (see e.g. Arkilic 2021), the non-resident population is deemed an important tool for advancing Turkish national interests abroad and to shore up support at home (c.f. Aydemir and Vermeulen 2023 in this special issue; Baser 2017; Yener-Roderburg 2020). Naturally, not all diaspora engagement efforts are exclusively geared towards the second generation. Improved access to administrative and consular services, or measures to facilitate external voting, benefit primarily first-generation migrants (Baser and Ozturk 2020). However, institutions like the *Yunus Emre Institutes*, or the “Presidency for Turks Abroad and Related Communities” (YTB) (Akçapar and Aksel 2017), which are directly tasked with engaging the Turkish diaspora, carry out activities—including cultural, or language classes—especially geared towards diaspora youth. The importance attributed to the second generation becomes apparent on the official YTB website, where the role and significance of cultural and language skills for young diasporans is stressed:

It is very important for our young people to be successful individuals in the countries where they live, protect their cultural values and pass them to the next generation. Our young people that understand the cultural values of the communities they live and are equipped with their own historical and cultural bounty, will contribute more to both countries (Presidency for Turks Abroad and Related Communities 2021).

In pursuit of this mission, the YTB organizes, with support of the Ministry of Youth and Sport, youth camps in Turkey for young diasporans (Bozkurt 2018). During the Covid-19 pandemic, the YTB sought to leverage alternative channels to reach out to diaspora youth “by using existing digitalized spaces such as Facebook, Instagram, YouTube, and Twitter, while also introducing new digitized formats such as digital concerts ‘*Dijital Konserler’* by famous diaspora artists” (Böcü and Baser 2020). Overall, we can clearly see how Turkey has embraced and tries to include youth it considers part of its diaspora.

Eritrea has a sizable diaspora due to its history of political violence and repression. The regime is actively engaging its diaspora, including many young regime supporters born outside the country. In June 2016, when thousands of Eritreans demonstrated in Geneva against what they saw as a flawed UN report criticizing Eritrea’s human rights record, young Eritreans were both among the speakers and protestors (TesfaNews 2016). The Young People’s Front for Democracy and Justice (YPFDJ) represents the exile youth branch of the PFDJ: the only legal political party since the country’s independence in 1991. The YPFDJ’s annual conference is deemed crucial for strengthening diaspora youth’s identification with the homeland. The conference serves as a meeting space and educates participants about the country’s history through seminars and lectures. Thus, the YPFDJ strives to socialize the young diaspora into remembering Eritrea’s traumatic and difficult past and instilling a sense of pride of the achievement of independence (Graf 2018). As true patriots, the generation growing up abroad is urged to support the “homeland” economically through a 2% diaspora tax and to help defend Eritrea’s reputation, as the country is internationally criticized for its poor human rights records (see e.g. Hirt 2021).

Morocco engages its young diaspora through various strategies. Cultural centres offer language training and cultural programmes, and the state organizes summer camps for children as well as so-called *Summer Universities* that aim to “preserve the national identity of the new generation of Moroccans living abroad” (quoted in: Mahieu 2019: 679). Every year, between 100 and 300 diasporans attend these all-expenses-paid visits to their ancestral homeland. The trips are intended to bolster transnational ties and increase the sense of identification with and loyalty to Morocco, by giving participants a personal experience that cannot be achieved through cultural or language classes. However, Mahieu (2019), in her study of the Summer Universities, shows that participants are not just passive recipients of the narrative of Morocco as a successful country, but critical agents who witness and interpret instances of corruption, poor organization and lack of freedom of speech within the programme as an illustration of the shortcomings of Morocco as a country. Reaching out to and including the second-generation/young diaspora is, thus, much less straightforward than authoritarian states may believe. Travelling “back home”, the second/young generation may not only discover the homeland, but also their otherness in relation to the local population—and perhaps the authoritarian state as well.

### How do homeland regimes exclude the second-generation diaspora?

The second-generation or young diaspora is not merely a target for or recipient of homeland states’ attempts to engage them as patriots or to co-opt them but may also criticize issues such as human rights violations. Their more secure situation, physically removed from the “homeland,” makes activism more feasible, not least due to factors such as citizenship and a more established position in their country of residence than their parents. This makes them particularly well-situated to mobilize and voice direct critique. While some youth organizations and diaspora activists might support authoritarian homeland regimes, there are numerous campaigns and initiatives through which this young generation joins forces to oppose these regimes. Logically, this turns the second generation into a potential threat, which forces authoritarian homeland regimes to devise strategies of exclusion or repression.

Ramzan Kadyrov, head of the Chechen Republic, is known for carrying out his own global strategy of repression against oppositional actors. In a 2016 televised speech, he warned regime detractors abroad:

I know all the sites, I know all the youth who live in Europe, every Instagram, Facebook, every social site, we record all of your words and we note them, we have all of your information, who, what, we know it all. This modern age and technology allow us to know everything and we can find any of you so don’t make it worse for yourselves (Schenkkan and Linzer 2021).

The formulation “all the youth” indicates that also those growing up outside of Chechnya are counted among those whom the regime either characterizes as good and supportive Chechens, or as traitors who are to be punished and repressed (Schenkkan and Linzer 2021).

In April 2021, Rwandan Minister of Justice, Busingye Johnston, tweeted that: “At the heart of the resurgence of genocide denial is a fringe of young, educated descendants of genocide perpetrators, mobilizing politically around JAMBO ASBL, an association, based in Belgium, of offspring of genocide perpetrators” (Johnston 2021). This is a remarkable accusation against a group of young people of Rwandan background whose organization strives to draw attention to atrocities committed against Hutus in Rwanda and the Democratic Republic of Congo (DRC). For the Rwandan government, fighting impunity and genocide denial is an important policy priority, but also—critics argue—a way to silence its opposition (Jansen 2014). While first-generation Hutu diasporans can be somewhat credibly accused of being genocide perpetrators when voicing anti-government critique, those who were either children or born after 1994 cannot be silenced in the same way. Therefore, they may be seen as even more threatening (Orjuela 2020). As indicated, the Rwandan government has attempted to include this group through, for instance, homeland visits. Unlike first-generation diaspora oppositional figures, physical attacks seem to be less of a concern for critics from the second generation—even though some have received threats (JamboNews 2020). Instead, the regime may rely on surveillance—as discussed in the introduction—or verbal attacks against them. Here, the Rwandan government’s campaign against the inclusion of young diaspora Rwandan and lawyer, Laure Uwase, in a special committee at the Belgian Chambers to examine the country’s colonial past in 2020, is yet another illustrative example (The New Times 2020).

While Turkey has, as noted above, expanded its outreach to the Turkish diaspora, this inclusion is highly selective. To some degree, it could be argued that the APK-led government actively chooses its diaspora—including some groups while simultaneously excluding others. Already during the 1980s and 1990s, there was a similar dynamic at play, when some diasporans were considered *good* or “pro-state” while others, for example Kurds, were considered *bad* or “anti-state” (Arkilic 2021). Nowadays, the AKP seems to conceptualize its diaspora to a large extent along the lines of Sunni-Turks, while others, like Kurds, Assyrians, or supporters of the outlawed Gülen movement, are neglected, excluded or outright considered enemies. A recent Freedom House (Schenkkan and Linzer 2021) report on transnational repression, and Baser and Ozturk (2020) outline some of the mechanisms, techniques and practices used to surveil, silence, intimidate or prosecute those whom the Erdogan regime deems undesirable. The German domestic intelligence service (*Bundesamt für Verfassungsschutz* [BfV] 2020) noted, for instance, in a recent report that Turkish intelligence services maintain a large network of informants in Turkish diaspora communities. Additionally, the Turkish regime seems to make extensive use of *red notices* in the Interpol system to harass or intimidate both real and perceived opponents. There have even been reports of illegal renditions of oppositional figures from third countries (Schenkkan and Linzer 2021). These practices seem, however, to be far less widespread in the context of second-generation diasporans. Here, an exclusion by omission appears to be the most prevalent tactic for those who are deemed either undesirable or unworthy of being included.

When revisiting the tactics and practices authoritarian regimes employ to repress or control their extraterritorial subjects, we find that only some are used against the generation that grew up in the diaspora. Techniques, such as surveillance, online intimidation, or coercion by proxy, may be used to target this generation. The aforementioned examples from Rwanda and Chechnya suggest that state officials—at least occasionally—voice accusations against young/second-generation critics in the diaspora. In theory, authoritarian states could just as well use physical attacks against this generation, but as far as we know, this is exceedingly rare. Whether this means that these individuals are seen as less threatening, or if attacking them would involve higher risks for authoritarian states, is worthy of further investigation. Moreover, the second-generation migrants’ relatively more secure position probably saves them from being a target of renditions or unlawful deportations (c.f. Schenkkan and Linzer 2021). Other techniques, such as passport cancellations or the denial of consular services, are, similarly, not quite as relevant when the targets are not citizens. However, in the Eritrean case, even non-citizens who have grown up and live outside the country can be pressured to support the homeland state. When paperwork is needed to, for instance, fulfil the wish of a deceased first-generation migrant to be buried in the homeland, or to resolve inheritance or land matters, authorities may demand that even grandchildren in the diaspora pay a retroactive diaspora tax (see e.g. Hirt and Saleh Mohammad 2018).

## Conclusions

Taking its point of departure in the burgeoning literature on the increasingly transnational nature of authoritarianism and the varied ways in which non-democratic regimes seek to engage, co-opt, control, or repress their extraterritorial populations, this article sought to draw attention to a particular group that has so far been largely neglected in these debates: second-generation diasporans. By teasing out the specific positionalities of diaspora youth, we have shown how certain inter-generational differences might complexify authoritarians’ efforts to selectively include or exclude what they see as their extraterritorial populations. Some of the strategies and practices that have proven efficacious in the context of first-generation migrants, like direct threats, renditions or even assassinations, are not nearly as feasible or effective when second-generation diasporans are concerned. While their skills and special position make them a highly interesting target group for engagement and co-optation, it also makes them less susceptible to certain more exclusionary practices.

The contribution of this article has been chiefly conceptual: to open a scholarly debate on the ways in which authoritarian regimes and their second/young generation diaspora interact. The realization that diaspora youth have emerged as a key target group for authoritarian states opens a host of new avenues for further research. For one, further in-depth empirical investigations into the motivations and strategies of authoritarian governments geared towards this group are warranted. At the same time, one ought to pay closer attention to the ways in which the (often democratic) countries of residence relate and react to authoritarians' efforts to engage with their young diasporas. Then, there is a clear need for studies inquiring into this group’s special positionality as they either support or challenge the governments of their ancestral homelands. Moreover, it will be fruitful to pay closer attention to the ways in which the second generation responds to these in- and exclusionary efforts. The second-generation or young diaspora is highly diverse, and future studies ought to acknowledge that the line between regime supporters and critics may sometimes be blurred: even those participating in activities supportive of an authoritarian regime may express critique or engage in resistance. Clearly, diaspora youth is not passive recipients of strategies of authoritarian regimes or, for that matter, the opposition. Understanding the complexities of how people of different ages and generations mobilize for or against authoritarian regimes and how these regimes seek to control or involve different sections of their diaspora will be increasingly important. What is at stake is the safety, freedom, and agency of individuals in the diaspora, but also broader development in both migrant-sending authoritarian states and the countries that migrants and their children inhabit.

## Footnotes

1 We define “diaspora” broadly as people residing outside of what they (or their country of origin) consider their homeland. We recognize, however, that diasporic identities are constructed and that diasporas are the result of mobilization processes (see e.g. Brubaker 2005; Sökefeld 2006).

2 This article defines the *second-generation diaspora* broadly as persons who grew up in a country their parents migrated to. The partly overlapping category *diaspora youth* is often used by both states and diasporans themselves, and thus, we employ that too when relevant (see e.g. Dahinden et al. 2021; Loizos 2007).

## References

[CR1] Abramson Y (2017). Making a homeland, constructing a diaspora: The case of Taglit-Birthright Israel. Political Geography.

[CR2] Adamson F (2020). Non-state authoritarianism and diaspora politics. Global Networks.

[CR3] Akçapar ŞK, Aksel DB (2017). Public diplomacy through diaspora engagement: The case of Turkey. Perceptions: Journal of International Affairs.

[CR4] Arkilic A (2021). Explaining the evolution of Turkey’s diaspora engagement policy: A holistic approach. Diaspora Studies.

[CR5] Aydemir N. and F. Vermeulen. 2023. Political preferences across a transnational space: interviews with dual citizens of the Netherlands and Turkey. *European Political Science*10.1057/s41304-022-00410-9

[CR6] Baeza C, Pinto P (2016). ‘Building support for the Asad regime: the Syrian diaspora in Argentina and Brazil and the Syrian uprising‘. Journal of Immigrant & Refugee Studies.

[CR7] Barwick C, Beaman J (2019). Living for the neighbourhood: Marginalization and belonging for the second-generation in Berlin and Paris. Comparative Migration Studies.

[CR8] Baser B, Ozturk AE (2020). Positive and negative diaspora governance in context: From public diplomacy to transnational authoritarianism. Middle East Critique.

[CR9] Baser, B. 2017. Turkey’s ever-evolving attitude-shift towards engagement with its diaspora, *emigration and diaspora policies in the age of mobility,* Springer, Cham.

[CR10] Betts A, Jones W (2016). Mobilizing the Diaspora.

[CR11] Bloch A (2018). Talking about the past, locating it in the present: the second generation from refugee backgrounds making sense of their parents. narratives, narrative Gaps and Silences. Journal of Refugee Studies.

[CR12] Bloch A, Hirsch S (2018). Inter-generational transnationalism: The impact of refugee backgrounds on second generation. Comparative Migration Studies.

[CR13] Böcü G. and B. Baser. 2020. ‘Authoritarian Home States and Diaspora Youth in Europe: Insights From the Turkish Case, *DIASCON*’ available at: https://www.diascon.eu/reports/blog/authoritarian-home-states-and-diaspora-youth-in-europe-insights-from-the-turkish-case/, Accessed 29 August 2022.

[CR14] Bozkurt A. 2018. *Turkey’s diaspora agency is building Erdoğan’s proxies abroad*, 2018, available at: https://www.turkishminute.com/2018/10/31/opinion-turkeys-diaspora-agency-is-building-erdogans-proxies-abroad/, Accessed 29 August 2022.

[CR15] Brand LA (2010). Authoritarian states and voting from abroad: North African experiences. Comparative Politics.

[CR16] Brinkerhoff JM (2019). Diasporas and public diplomacy: Distinctions and future prospects. The Hague Journal of Diplomacy.

[CR17] Brubaker R (2005). The ‘diaspora’diaspora. Ethnic and Racial Studies.

[CR18] Bruland S (2012). Nationalism as meaningful life projects: Identity construction among politically active Tamil families in Norway. Ethnic and Racial Studies.

[CR19] Bundesamt für Verfassungsschutz (BfV). 2020.*Verfassungsschutzbericht 2019, available at *https://www.verfassungsschutz.de/SharedDocs/publikationen/DE/verfassungsschutzberichte/2020-07-verfassungsschutzbericht-2019-fakten-und-tendenzen-kurzzusammenfassung.html*, Accessed 29 August 2022* .

[CR20] Conrad B (2006). A culture of War and a culture of exile: Young Eritreans in Germany and their relations to Eritrea. La Revue Européenne Des Migrations Internationales.

[CR21] Dahinden J, Fischer C, Menet J (2021). Knowledge production, reflexivity, and the use of categories in migration studies: Tackling challenges in the field. Ethnic and Racial Studies.

[CR22] Fouron G. and N.Glick- Schiller. 2002. ‘The Generation of Identity: Redefining the Second Generation within a Transnational Social Field’, in Levitt, P. & Waters, M. C. (eds), *The Changing Face of Home: The Transnational Lives of the Second Generation*. Russel Sage Foundation.

[CR23] Frantz E, Kendall-Taylor A (2014). ‘A dictator’s toolkit: Understanding how co-optation affects repression in autocracies‘. Journal of Peace Research.

[CR24] Gerschweski J (2013). The three pillars of stability: Legitimation, repression, and co-optation in autocratic regimes. Democratization.

[CR25] Glasius M (2017). Extraterritorial authoritarian practices: A framework. Globalizations.

[CR26] Graf S (2018). Politics of belonging and the Eritrean diaspora youth: Generational transmission of the decisive past. Geoforum.

[CR27] Hirsch M (2008). The generation of postmemory. Poetics Today.

[CR28] Hirt N (2021). Eritrea’s chosen trauma and the legacy of the martyrs: The impact of Postmemory on political identity formation of second- generation diaspora eritreans. Africa Spectrum.

[CR29] Hirt N, Saleh Mohammad A (2018). ‘By way of patriotism, coercion, or instrumentalization: How the Eritrean Regime makes use of the diaspora to stabilize its rule‘. Globalizations.

[CR30] Horst C (2018). Making a difference in Mogadishu? Experiences of multi-sited embeddedness among diaspora youth. Journal of Ethnic and Migration Studies.

[CR31] Huang W-J (2021). Influence of transnational leisure on diaspora tourism among contemporary migrants. Journal of Travel Research.

[CR32] JamboNews. 2020. Carine Kanimba: “In the name of my father, Paul Rusesabagina”—hero of the movie Hotel Rwanda, available at: https://www.jambonews.net/en/actualites/20201231-carine-kanimba-in-the-name-of-my-father-paul-rusesabagina-hero-of-the-movie-hotel-rwanda/, Accessed 29 August 2022

[CR33] Jansen Y-O (2014). Denying genocide or denying free speech—A case study of the application of Rwanda’s genocide denial laws. Northwestern Journal of International Human Rights.

[CR34] Johnston B. 2021.At the heart of the resurgence of genocide denial is a fringe of young, educated descendants of genocide perpetrators, *Twitter*, available at: https://twitter.com/BusingyeJohns/status/1381834919178084352, Accessed 29 August 2022.

[CR35] Lamblin C, Beaugrand C, Geisser V (2018). To what extent can the January 25 Revolution be seen as a “bifurcation” in the life stories of Egyptian migrants in France?. Diasporic Social Mobilization and Political Participation during the Arab Uprisings.

[CR36] Lee H (2011). Rethinking transnationalism through the second generation. The Australian Journal of Anthropology.

[CR37] Levitt P (2009). Roots and routes: Understanding the lives of the second generation transnationally. Journal of Ethnic and Migration Studies.

[CR38] Loizos P (2007). ’Generations’ in forced migration: Towards greater clarity. Journal of Refugee Studies.

[CR39] Lührmann A, SF. Maerz, S. Grahn, N. Alizada, L. Gastaldi, S. Hellmeier, G. Hindle, and SI. Lindberg. 2020. ‘Autocratization Surges - Resistance Grows: Democracy report 2020’, *Varieties of Democracy Institute* (V-Dem).

[CR40] Lyons T, Mandaville P. 2012. ‘*Politics from Afar: Transnational Diasporas and Networks*‘ Hurst & Company (Publishers)

[CR41] Mahieu R (2019). ‘‘We’re not coming from Mars; we know how things work in Morocco!’How diasporic Moroccan youth resists political socialisation in state-led homeland tours‘. Journal of Ethnic and Migration Studies.

[CR42] Mencutek ZS, Baser B (2018). Mobilizing diasporas: Insights from Turkey’s Attempts to Reach Turkish citizens abroad. Journal of Balkan and near Eastern Studies.

[CR43] Moss D (2016). Transnational repression, diaspora mobilization, and the case of the Arab spring. Social Problems.

[CR44] Moss D., M. Michaelsen, and G. Kennedy. 2022. ‘Going after the family: Transnational repression and the proxy punishment of Middle Eastern diasporas’, *Global Networks* advance online publication 10 May. 10.1111/glob.12372.

[CR45] Müller-Funk L (2016). Diaspora mobilizations in the Egyptian (post) revolutionary process: Comparing transnational political participation in Paris and Vienna. Journal of Immigrant & Refugee Studies.

[CR46] OCCRP. 2021. Israeli Spy Tech Used Against Daughter of Man Who Inspired “Hotel Rwanda”, available online: https://www.occrp.org/en/the-pegasus-project/israeli-spy-tech-used-against-daughter-of-man-who-inspired-hotel-rwanda?fbclid=IwAR3gEj63GAL6c9kWKG6jSJI-lOZC6pb_6UcXnINdxng8H9P7c4L5_KEuxSQ&s=09, Accessed 29 August 2022.

[CR47] Orjuela C (2008). Distant warriors, distant peace workers? Multiple diaspora roles in Sri Lanka’s violent conflict. Global Networks.

[CR48] Orjuela C (2020). Passing on the torch of memory: Transitional justice and the transfer of diaspora identity across generations. International Journal of Transitional Justice.

[CR49] Østergaard-Nielsen EK (2001). ‘Transnational political practices and the receiving state: Turks and Kurds in Germany and the Netherlands. Global Networks.

[CR50] Pedroza L, Palop-García P (2017). Diaspora policies in comparison: An application of the emigrant policies index (EMIX) for the Latin America and Caribbean region. Political Geography.

[CR51] Presidency for Turks Abroad and Related Communities. 2021. *Anatolia Weekend Schools*, 2021, available at: https://www.ytb.gov.tr/en/abroad-citizens/anatolia-weekend-schools, Accessed 29 August 2022

[CR52] Ragazzi F (2014). A comparative analysis of diaspora policies. Political Geography.

[CR53] Rashkova ER (2020). The party abroad: a new modus operandi for political parties. Parliamentary Affairs.

[CR54] Sá AL, Rodrigues Sanches E (2021). The politics of autocratic survival in Equatorial Guinea: Co-optation, restrictive institutional rules, repression, and international projection. African Affairs.

[CR55] Saey S, Skey M (2016). The politics of trans-national belonging: A study of the experiences of second-generation Egyptians during a period of socio-political change in Egypt. Migration Studies.

[CR56] Schenkkan N. and I. Linzer. 2021. Out of sight, not out of reach: the global scale and scope of transnational repression, Freedom House

[CR57] Soehl T, Waldinger R (2012). Inheriting the homeland? Intergenerational transmission of cross-border ties in migrant families. American Journal of Sociology.

[CR58] Sökefeld M (2006). Mobilizing in transnational space: A social movement approach to the formation of diaspora. Global Networks.

[CR59] TesfaNews. 2016. Eritreans Staged Mass Demonstration in Geneva Denouncing COI Report, available online: https://tesfanews.net/eritreans-staged-mass-deneva-demonstration-in-geneva-denouncing-coi/, Accessed 29 August 2022

[CR60] The New Times. 2020. Outrage as genocide denier is chosen expert on Belgian colonial role in Rwanda, available online: https://www.newtimes.co.rw/news/outrage-genocide-denier-chosen-expert-belgian-colonial-role-rwanda, Accessed 29 August 2022

[CR61] Tsourapas G (2015). Why do states develop multi-tier emigrant policies? Evidence from Egypt. Journal of Ethnic and Migration Studies.

[CR62] Tsourapas G (2016). Nasser’s educators and agitators across al-Watan al-‘Arabi: Tracing the foreign policy importance of Egyptian regional migration 1952–1967. British Journal of Middle Eastern Studies.

[CR63] Tsourapas G (2021). Global autocracies: Strategies of transnational repression, legitimation, and co-optation in world politics. International Studies Review.

[CR64] Turner S (2013). Staging the Rwandan diaspora. African Studies.

[CR65] Umpierrez de Reguero S. and M.L. Jakobson. 2023. Explaining support for populists among external voters: between home and host Country. *European Political Science* 22(1)

[CR66] Umpierrez de Reguero S. and J. Peltoniemi. 2023. Introduction: Non-Residents’ Participation in the Homeland Arena from a European Perspective European Political Science 22(1)

[CR67] Wellman EI (2021). Emigrant inclusion in home country elections: Theory and evidence from sub-Saharan Africa. American Political Science Review.

[CR68] Wintrobe R (2000). The political economy of dictatorship.

[CR69] Yener-Roderburg, I.Ö. 2020. Top-down satelites and bottom-up alliances: The case of AKP and HDP in Germany, in Kernalegenn, T. & Van Haute, È. (eds), *Political Parties Abroad*. Routledge.

[CR70] Zhou M (1997). Segmented assimilation: Issues, controversies, and recent research on the new second generation. International Migration Review.

